# Supplementary material for: Functionally distinct roles for eEF2K in the control of ribosome availability and p-body abundance
Source: Nat Commun. 2021 Nov 23;12:6789. doi: 10.1038/s41467-021-27160-4 (PMC8611098; doi:10.1038/s41467-021-27160-4)
Supplement: Supplementary file 1 — Supplementary Information [file 41467_2021_27160_MOESM1_ESM.pdf]

Supplementary Information

Functionally distinct roles for eEF2K in the control of ribosome availability and p-body abundance

Patrick R. Smith[1*], Sarah Loerch[2,3*], Nikesh Kunder[1], Alexander D. Stanowick[1], Tzu-Fang Lou[1], and Zachary T. Campbell[1,4]

[1] The University of Texas at Dallas, Department of Biological Sciences, Richardson, TX
[2] Janelia Research Campus, Howard Hughes Medical Institute, Ashburn, VA
[3] University of California, Santa Cruz, Department of Chemistry and Biochemistry, Santa Cruz, CA
[4] The Center for Advanced Pain Studies (CAPS), University of Texas at Dallas, Richardson, TX
* These authors contributed equally to this work

Correspondence:
Zachary T. Campbell, Ph. D.
Department of Biological Sciences
800 W. Campbell Road, RL10 BSB 12.510
Richardson, TX, 75080
Phone: 972-883-4186
E-mail: Zachary.Campbell@utdallas.edu

Number of figures: 7
Number of tables: 1
Number of references: 1

Supplementary figures:

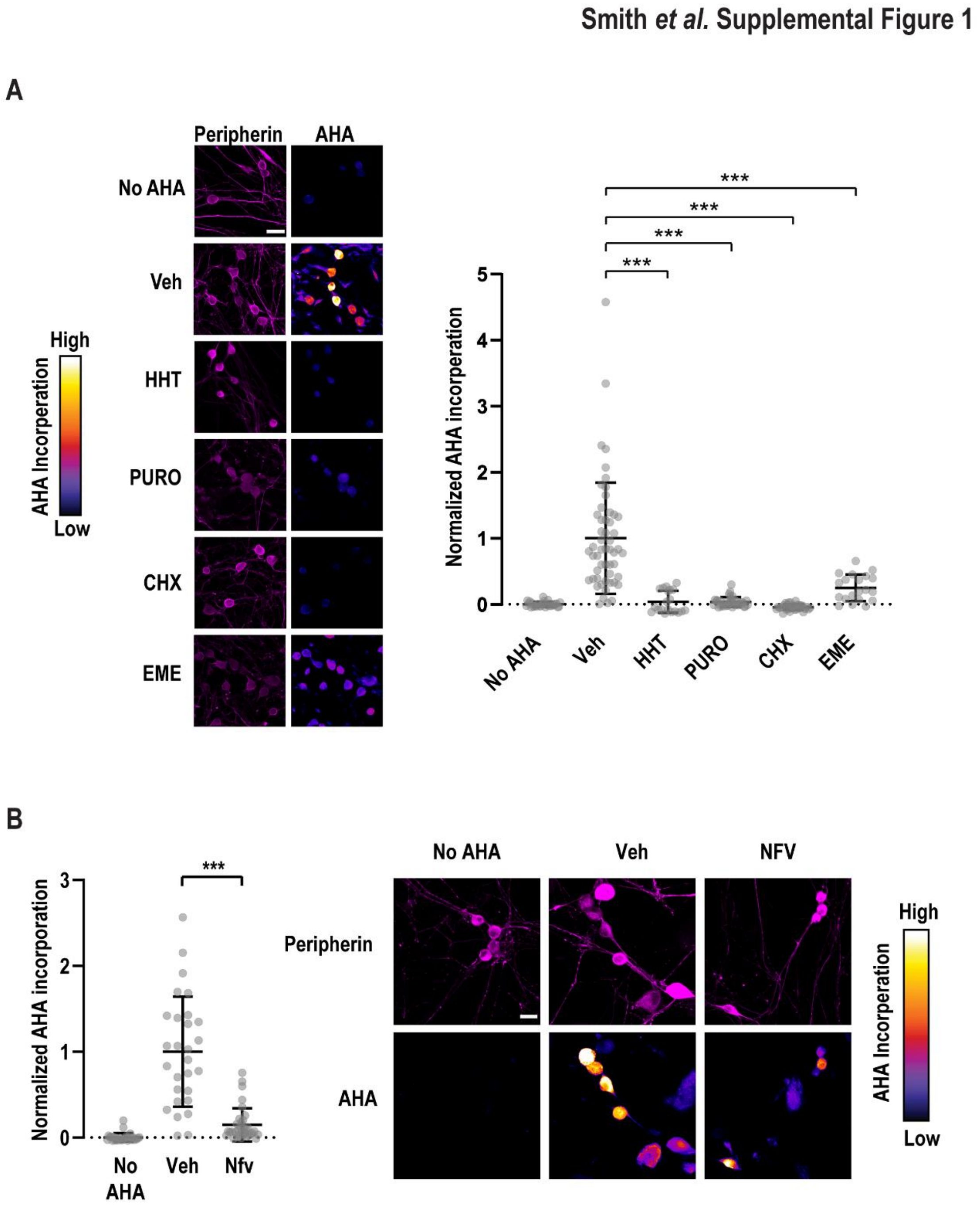

Figure S1 – Quantification of nascent translation

(A) Primary DRG cultures from WT mice were treated with vehicle, homoharringtonin (HHT), puromycin (PURO), cycloheximide (CHX), or emetine (EME) for 1 hour and subjected to a 30-minute pulse of AHA to label nascent peptides. Cells were subjected to FUNCAT and peripherin immuno-labeling and imaged via confocal microscopy. To quantify the baseline, a control group without AHA was also imaged. (A, left) Representative confocal images. Scale bar = 30 µm. (B, right) Quantification of relative AHA incorporation in peripherin-positive cells. For No AHA, Veh, CHX, HHT, PURO, and EME, n = 30, 53, 20, 36, 33, and 20 cells, respectively. Bars indicate mean ±SD P-values determined by one-way ANOVA. Veh vs. HHT $p < 0.0001$, Veh vs. PURO $p < 0.0001$, Veh vs. CHX $p < 0.0001$, Veh vs. EME $p < 0.0001$.

(B) Primary DRG cultures from homozygous eEF2K KO mice were treated with vehicle (Veh) or nelfinavir (NFV) for 1 hour and subjected to a 30 min pulse of AHA. Cultures were then used for FUNCAT and peripherin immuno-labeling. (B, left) Quantification of AHA incorporation in peripherin-positive cells. For No AHA, Veh, and NFV n = 28, 28, and 34 cells, respectively. Bars indicate mean ±SD. P-values determined by one-way ANOVA. Veh vs. NFV $p < 0.0001$. (Right) Representative confocal images from FUCAT with eEF2K KO cells. Scale bar = 20 µm. Source data are provided as a Source Data file.

Smith et al. Supplemental Figure 2

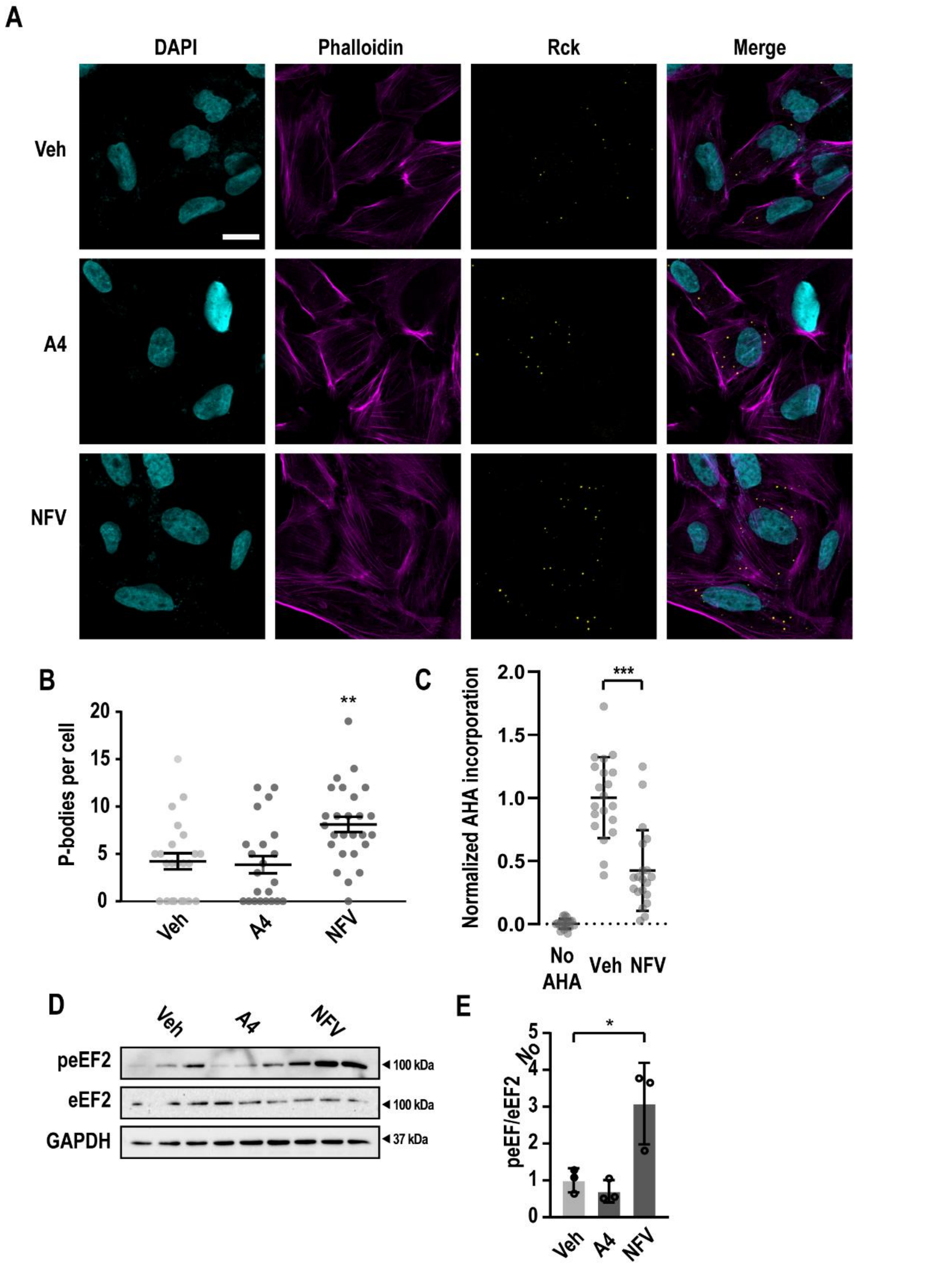

Figure S2 – P-bodies in cell lines are not insensitive to eEF2K modulation.

(A) Representative ICC image. U2OS cells were treated with vehicle (Veh), A484954 (A4), or nelfinavir (NFV) for 1 hour prior to fixation and ICC. Cells were labeled with phalloidin-TRITC (magenta) and Rck (yellow) was immuno-labeled to mark p-bodies. Nuclei were stained with DAPI. Scale bar = 30 µm

(B). Quantification of p-bodies corresponds to the sample groups in panel A. For Veh, A4, and NFV n = 23, 22, and 26 cells, respectively. The error bars represent mean ± S.E.M. P-values determined by one-way ANOVA. Veh vs. NFV p = 0.0032

(C) U2OS cells were treated with vehicle (Veh) or nelfinavir (NFV) as in (A), with the addition of a 30-min pulse of AHA. Samples were then used for FUNCAT assay. Quantification of mean AHA incorporation was normalized to signal from AHA-free cells, n = 20 cells. Bars indicate mean ±SD P-values determined by one-way ANOVA. Veh vs. NFV p < 0.0001.

(D) U2OS cells were treated as in (A) and used to generate lysates for immunoblots. Lysates were probed for p-eEF2, eEF2, and GAPDH (load control).

(E) Average p-eEF2/eEF2 signal from blots represented in (D). Error bars represent ±SD. n = 3 biological replicates P-values determined by one-way ANOVA. Veh vs. NFV p = 0.0176. Source data are provided as a Source Data file.

Smith et al. Supplemental Figure 3

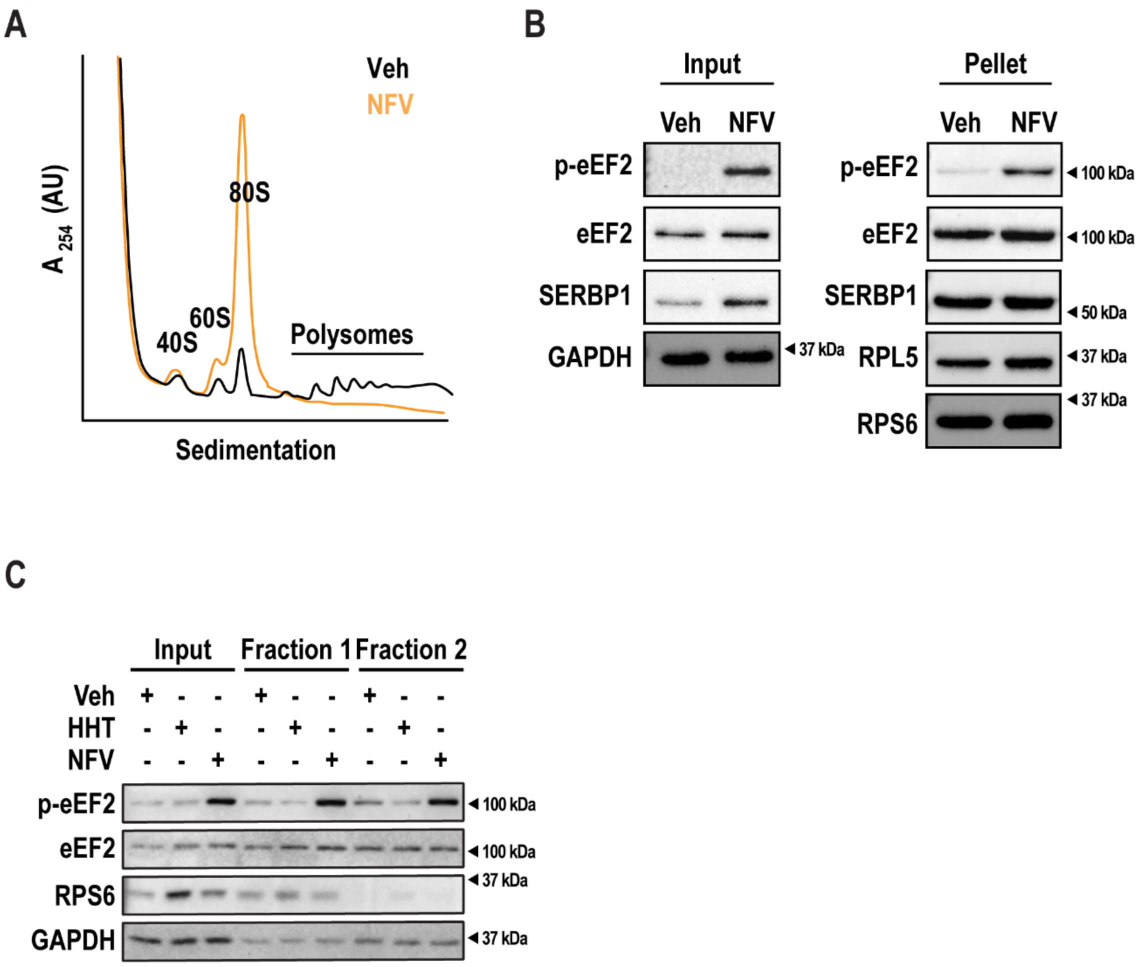

Figure S3 – p-eEF2 co-fractionates with ribosomes isolated for cryo-EM

(A) Representative polysome profiles following treatment with vehicle (black) or nelfinavir (orange). F11 cells were treated with either vehicle (Veh) or nelfinavir (NFV) for 1 hour. Cells were lysed and used to generate polysome profiles. Polysomes were not stabilized with an antibiotic.

(B) Representative immunoblots of ribosomes purified by sucrose cushion. Primary DRG neurons were treated with either vehicle (Veh) or nelfinavir (NFV) for 1 hour, followed 100 µM emetine for 5 minutes. Cells were lysed and loaded on 30% sucrose cushions before ultracentrifugation to pellet ribosomes. Immunoblots were performed using input and resuspended ribosome pellets in three technical replicates.

(C) Representative immunoblots from three biological replicates of ribosomes isolated from primary DRG by rapid SEC. Primary DRG cultures were treated with vehicle (Veh), homoharringtonine (HHT), or nelfinavir (NFV) for 1 hour, followed by a 5-minute treatment with

100 µM emetine to halt translating ribosomes. Lysates were generated and fractionated using S400 size-exclusion columns, into ribosome-containing fractions (Fraction1) and ribosome-free fractions (Fraction 2).

Smith et al. Supplemental Figure 4

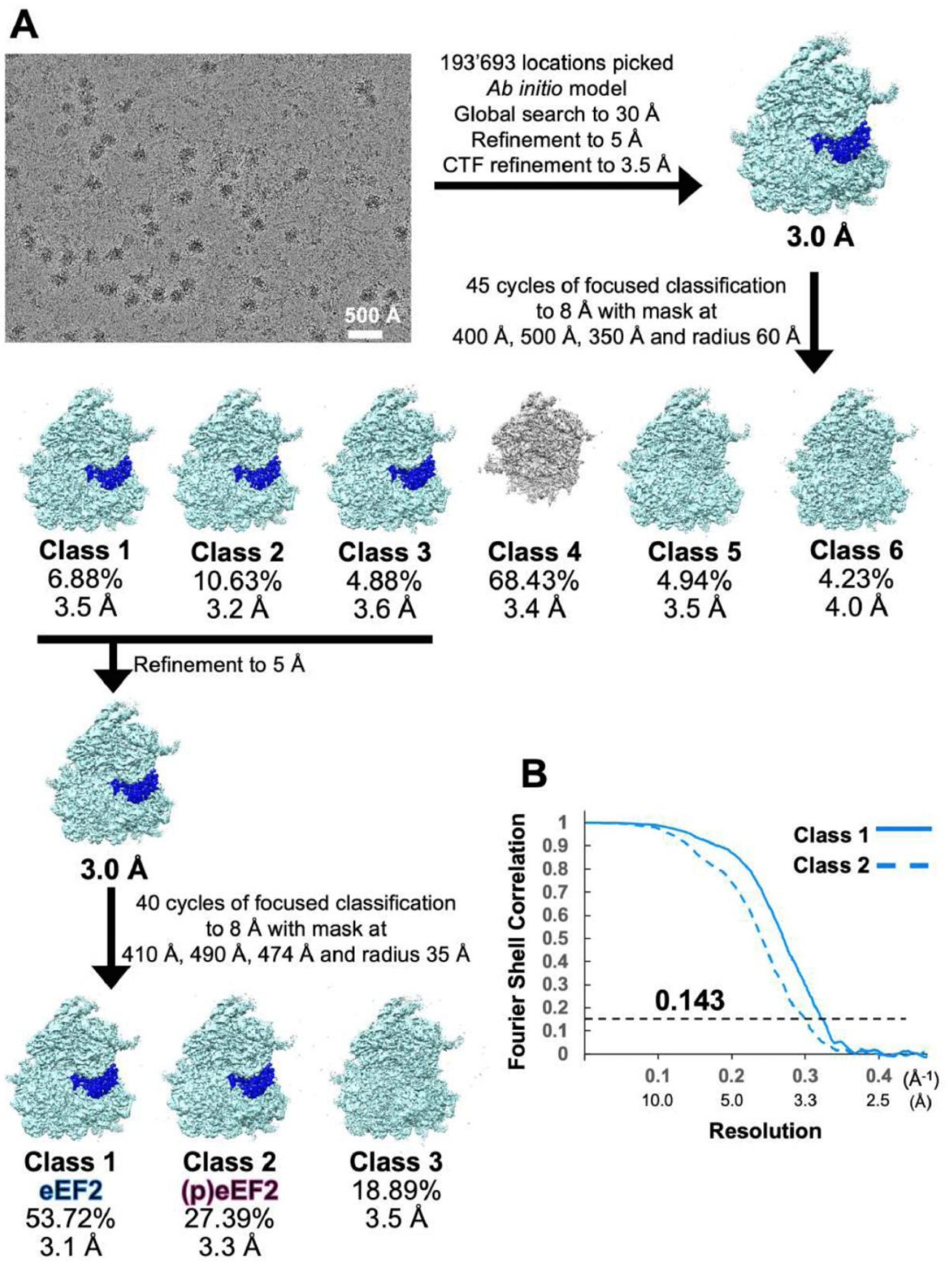

Figure S4 – Classification scheme of 80S mouse ribosomes isolated from DRG neurons. (A) Representative micrograph and classification workflow. The initial model was obtained *ab initio* and all particles were initially aligned to this reference. First, we classified into six classes using a spherical focus mask around the A-site, yielding three classes with eEF2 density, which we merged and aligned to a common reference. We then classified again into three classes using a smaller spherical focus mask encompassing domains I and II of eEF2, which yielded one class with eEF2, one class with what we interpreted as (p)eEF2, and one class without eEF2. (B) Fourier Shell Correlations of Classes I and II comprising ribosomes bound to eEF2 and (p)eEF2, respectively.

Smith *et al.* Supplemental Figure 5

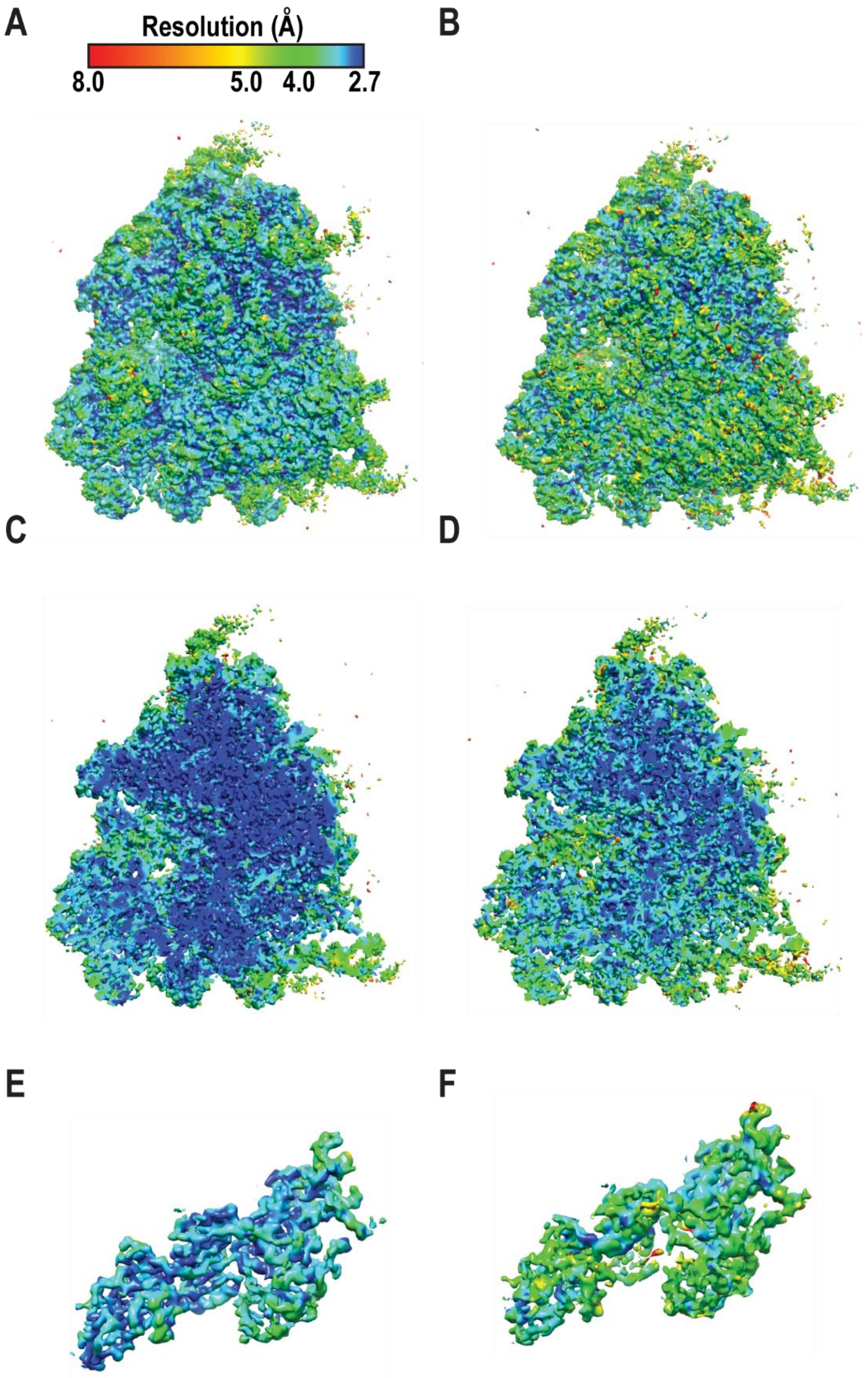

Figure S5 – Local resolution maps of class I (A, C, E) and class II (B, D, F). All maps were colored according to the estimated local resolution determined using Blocres [1]. The top row shows the view on the E/P/A sites, the middle row shows the cut-through view, and the bottom row shows eEF2 for classes I (left) and II (right), respectively.

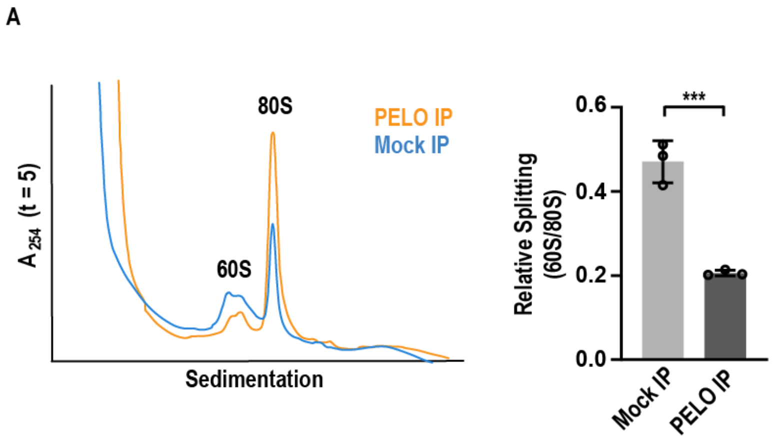

Figure S6 – Ribosome splitting is reduced by Pelo depletion

(A) F11 cells were treated with puromycin for 5 minutes and used to generate lysates for *in* vitro splitting reactions. Prior to initiating reactions, lysates were depleted of Pelota (PELO) via immunoprecipitation (or mock depleted). Reactions were then initiated as before by the addition of ATP (1 mM), GTP (1 mM), and eIF6 (5 µM), and incubated at 37°C for five minutes. Reactions were halted by cooling on ice before being used to generate polysome profiles. (A, left) Representative polysome profiles from *in vitro* splitting assays performed with mock-depleted (cyan) and Pelo-depleted (orange) lysates.

(A, right) Mean 60S/80S peak height ratios from 3 biological replicates. Error bars represent ± SD. P =0.001. Source data are provided as a Source Data file.

Smith et al. Supplemental Figure 7

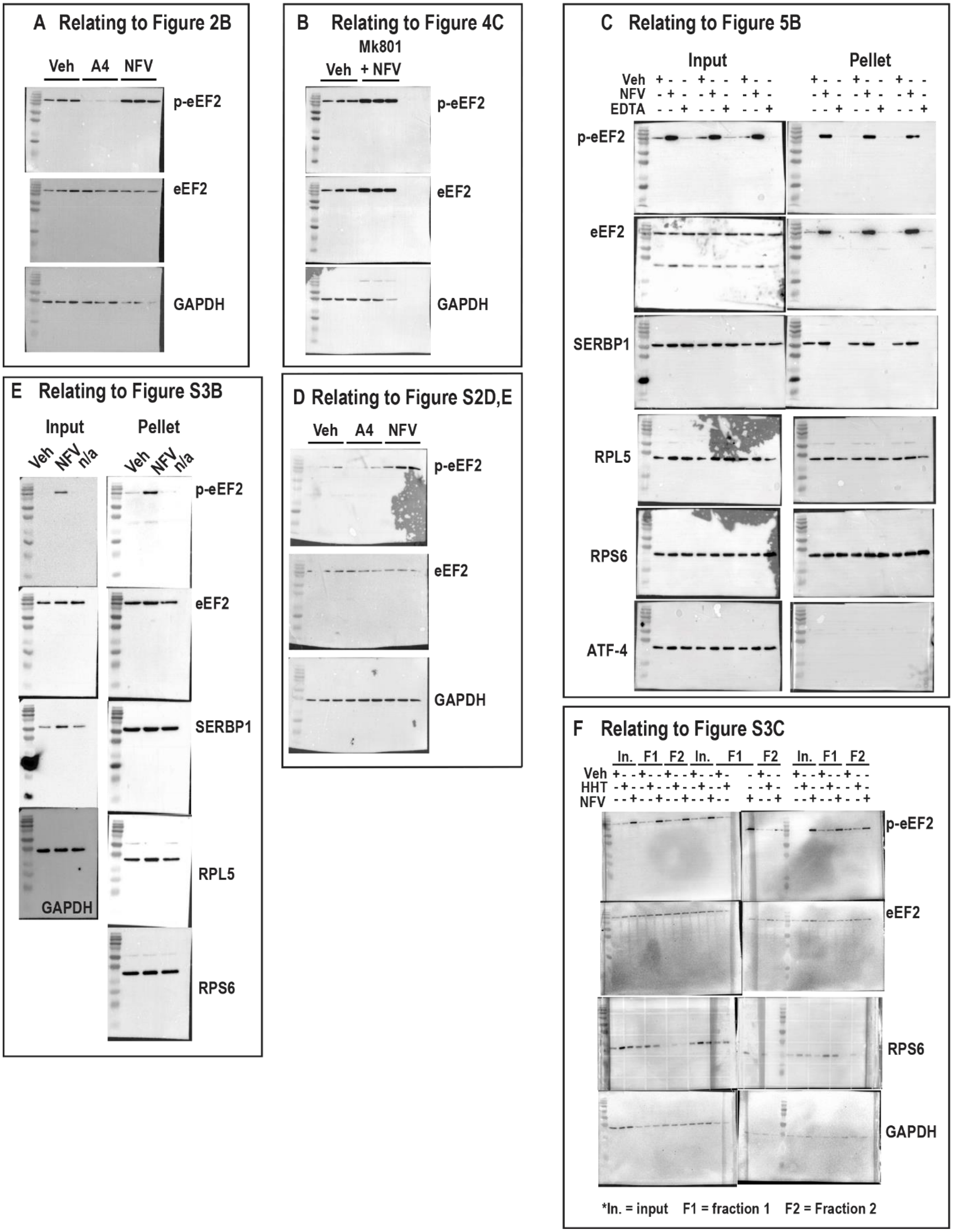

Figure S7 – Uncropped immunoblots

(A) Relating to Figure 2B

(B) Relating to Figure 4C

(C) Relating to Figure 5B

(D) Relating to Supplemental Figure S2D,E

(E) Relating to Supplemental Figure 3B

(F) Relating to Supplemental Figure 3C

Supplementary table:

Cryo-EM data collection, refinement, and validation statistics

	Class 1 (EMDB-23501) (PDB 7LS2)	Class 2 (EMDB-23500) (PDB 7LS1)
**Data collection and processing**		
Magnification	x 81,000	x 81,000
Voltage (kV)	300	300
Electron exposure (e–/Å^2)	75	75
Defocus range (μm)	-0.5 to -2.5	-0.5 to -2.5
Pixel size (Å)	1.06	1.06
Symmetry imposed	N/A	N/A
Initial particle images (no.)	193,794	193,794
Final particle images (no.)	23,297	11,878
Map resolution (Å)	3.1	3.3
FSC threshold	0.143	0.143
**Refinement**		
Initial model used (PDB code)	6ek0 and 6mtd	6ek0 and 6mtd
Model resolution (Å)	3.67	3.97
FSC threshold	0.5	0.5
Map sharpening *B* factor (Å^2)	0 to -90	0 to -90
Model composition		
Non-hydrogen atoms	227,130	227,065
Protein residues	12,862	12,855
Nucleotides	5,780	5,780
B factors (Å^2)		
Protein	143.52	157.59
Nucleotide	142.69	163.45
Ligand	131.24	153.76
R.m.s. deviations		
Bond lengths (Å)	0.002	0.02
Bond angles (°)	0.481	0.493
Validation		
MolProbity score	1.60	1.60
Clashscore	7.72	8.06
Poor rotamers (%)	1.16	1.08
Ramachandran plot		
Favored (%)	97.35	97.28
Allowed (%)	2.63	2.70
Disallowed (%)	0.02	0.02

Supplementary Reference:

1. Cardone G, Heymann JB, Steven AC One number does not fit all: mapping local variations in resolution in cryo-em reconstructions. J Struct Biol **184**:226–236. (2013)
