# Supplementary material for: Functionally distinct roles for eEF2K in the control of ribosome availability and p-body abundance
Source: Nat Commun. 2021 Nov 23;12:6789. doi: 10.1038/s41467-021-27160-4 (PMC8611098; doi:10.1038/s41467-021-27160-4)
Supplement: Supplementary file 3 — Reporting Summary [file 41467_2021_27160_MOESM3_ESM.pdf]

nature research

Corresponding author(s): Zachary Campbell

Last updated by author(s): Apr 1, 2021

Reporting Summary

Nature Research wishes to improve the reproducibility of the work that we publish. This form provides structure for consistency and transparency in reporting. For further information on Nature Research policies, see our Editorial Policies and the Editorial Policy Checklist.

Statistics

For all statistical analyses, confirm that the following items are present in the figure legend, table legend, main text, or Methods section.

n/a	Confirmed	
☐	☒	The exact sample size (*n*) for each experimental group/condition, given as a discrete number and unit of measurement
☒	☐	A statement on whether measurements were taken from distinct samples or whether the same sample was measured repeatedly
☐	☒	The statistical test(s) used AND whether they are one- or two-sided<br>*Only common tests should be described solely by name; describe more complex techniques in the Methods section.*
☐	☒	A description of all covariates tested
☐	☒	A description of any assumptions or corrections, such as tests of normality and adjustment for multiple comparisons
☐	☒	A full description of the statistical parameters including central tendency (e.g. means) or other basic estimates (e.g. regression coefficient) AND variation (e.g. standard deviation) or associated estimates of uncertainty (e.g. confidence intervals)
☐	☒	For null hypothesis testing, the test statistic (e.g. *F*, *t*, *r*) with confidence intervals, effect sizes, degrees of freedom and *P* value noted<br>*Give P values as exact values whenever suitable.*
☒	☐	For Bayesian analysis, information on the choice of priors and Markov chain Monte Carlo settings
☒	☐	For hierarchical and complex designs, identification of the appropriate level for tests and full reporting of outcomes
☐	☒	Estimates of effect sizes (e.g. Cohen's *d*, Pearson's *r*), indicating how they were calculated

Our web collection on statistics for biologists contains articles on many of the points above.

Software and code

Policy information about availability of computer code

Data collection	Fiji - used for analysis of fluorescence microscopy images<br>Olympus FluoView - used for batch Z projection of confocal images<br>ImageLab 6.0 - used for quantification of immunoblot data<br>SerialEM - used to collect cryo-EM data<br>GraphPad Prism 8 - data recording, analysis, and plot generation
Data analysis	cisTEM - used to process cryo-EM data. Version 2.0.0 (available on github)<br>Coot 0.9.3 - as integrated into CCPEM used for manual model building (open source)<br>Phenix 1.19.1-4122 used for model refinement (open source)

For manuscripts utilizing custom algorithms or software that are central to the research but not yet described in published literature, software must be made available to editors and reviewers. We strongly encourage code deposition in a community repository (e.g. GitHub). See the Nature Research guidelines for submitting code & software for further information.

Data

Policy information about availability of data

All manuscripts must include a data availability statement. This statement should provide the following information, where applicable:

- Accession codes, unique identifiers, or web links for publicly available datasets
- A list of figures that have associated raw data
- A description of any restrictions on data availability

Structural models have been deposited in the PDB under the accession codes 7LS2 (class I) and 7LS1 (class II). Cryo-EM maps have been deposited to the EMDB under the accession codes EMD-23501 (class I) and EMD-23500 (class II). Single-cell RNA-seq data generated by Usoskin et al. are accessible through Gene

Expression Omnibus, Accession code GSE59739 77,78. Source data for figures 1 – 4, 7, S1, S2, and S6 are provided with this paper. Uncropped blot images are provided in the supplement (Fig. S7).

Field-specific reporting

Please select the one below that is the best fit for your research. If you are not sure, read the appropriate sections before making your selection.

☒ Life sciences ☐ Behavioural & social sciences ☐ Ecological, evolutionary & environmental sciences

For a reference copy of the document with all sections, see nature.com/documents/nr-reporting-summary-flat.pdf

Life sciences study design

All studies must disclose on these points even when the disclosure is negative.

Sample size

Sample sizes for cell imaging experiments was similar to that of prior publications (e.g. DOI 10.1523/JNEUROSCI.4155-08.2008, DOI 10.1016/j.neulet.2013.09.048). Sample sizes for immunoblot and biochemical experiments was similar to that of prior publications (e.g. DOI 10.1038/s41467-021-21637-y, DOI 10.1038/s41467-020-15412-8, DOI 10.1371/journal.pbio.3000780)

For single particle cryo-EM, we collected a dataset encompassing 2,995 movies that yielded a dataset of 193,796 ribosome coordinates. A high-resolution reconstruction of a ribosome requires ~5000 images of a ribosome (particles). We collected sufficient data to determine the high-resolution structures of each eEF2-containing class (23,297 and 11,878, for classes 1 and 2, respectively).

Data exclusions

No data were excluded from analyses

Replication

We define technical replicates as those performed in parallel from the same original biological sample, and biological replicates as those performed using separate biological samples. For confocal imaging experiments, several individual cells were measured from at least two biological replicates to achieve an appropriate n. Biological replicates for immunoblots from total cell lysates and ribosome isolations were conducted in parallel. All polysome profile experiments were performed with three biological replicates. Overlayed representative polysome profiles were conducted in parallel. Reported data for confocal imaging and polysome profiles were derived from successfully replicated experiments.

Randomization

Randomization was not relevant to this study, as no in vivo or human studies were conducted

Blinding

No data in this study derived from subjective assessments by the experimenter. As such, blinding was not relevant to this study

Reporting for specific materials, systems and methods

We require information from authors about some types of materials, experimental systems and methods used in many studies. Here, indicate whether each material, system or method listed is relevant to your study. If you are not sure if a list item applies to your research, read the appropriate section before selecting a response.

Materials & experimental systems

n/a	Involved in the study	
☐	☒	Antibodies
☐	☒	Eukaryotic cell lines
☒	☐	Palaeontology and archaeology
☐	☒	Animals and other organisms
☒	☐	Human research participants
☒	☐	Clinical data
☒	☐	Dual use research of concern

Methods

n/a	Involved in the study	
☒	☐	ChIP-seq
☒	☐	Flow cytometry
☒	☐	MRI-based neuroimaging

Antibodies

Antibodies used

Anti-Rck, Rb polyclonal (1:1000) MBL PD009 Lot 032
Anti-Rck, Mm monoclonal (1:500) SCBT 376433 Lot J0317
Anti-Peripherin, Ck polyclonal (1:1000) Novus NBP1-0543
Anti-eEF2K Rb polyclonal (1:400) Invitrogen PA5-22175 Lot UH2831084A
Phalloidin-TRITC (1:200) Sigma P1951
Anti-p-eEF2 (T56), Rb polyclonal (1:1000) CST 2331S Lot 9
Anti-eEF2, Rb polyclonal (1:1000) CST 2332S Lot 7
Anti-SERBP1, Rb polyclonal Bethyl (1:1000) A303-938A Lot1
Anti-RPL5, Rb polyclonal Bethyl (1:1000) A303-933A
Anti-RPS6, Rb monoclonal (1:1000) CST 2217s Lot 10

	Anti-ATF-4, Rb monoclonal (1:1000) CST 11815S Lot 4<br>Anti-GAPDH, Rb polyclonal (1:10,000) Proteintech 10494<br>Anti-PELO, Mm polyclonal (12µg per 30µl protein A/G beads) SCBT 393418 Lot B0315<br>Gt-anti-Rb-Cy5 polyclonal (1:1000)Invitrogen A10523 Lot 1675037<br>Gt-anti-Ck-AlexaFluor488 (1:1000) polyclonal Invitrogen A11039 Lot 1869581<br>Gt-anti-Mm-Cy5 polyclonal (1:1000) Invitrogen A10524 Lot 1675775<br>Gt-anti-Rb-peroxidase (1:10,000) Invitrogen 32460 Lot QG220833
Validation	Anti-Rck, Rb readily detects RCK in fixed mouse cells (e.g. DOI 10.1523/JNEUROSCI.0104-08.2008, DOI 10.1038/s41598-018-30805-y)<br>Anti-Rck, Mm readily detects RCK in fixed human cells (e.g. 10.1038/s41467-019-08548-9)<br>Anti-Peripherin, Ck indicated by manufacturer for immunofluorescence detection of peripherin; specifically labels peripheral neurons in heterogenous primary cultures (e.g. DOI: 10.1111/bph.15646)<br>Anti-eEF2K, Rb indicated by manufacturer for immunofluorescence detection of eEF2K in fixed cells<br>Phalloidin-TRITC readily detects actin cytoskeleton of eukaryotic cells (e.g. DOI 10.1038/s41467-017-02449-5)<br>Anti-p-eEF2 (T56), Rb readily detects phospho-eEF2 (Thr56) in immunoblot (e.g. DOI 10.3389/fnmol.2019.00097)<br>Anti-eEF2, Rb readily detects eEF2 in immunoblot (e.g. DOI 10.3389/fnmol.2019.00097)<br>Anti-SERBP1, Rb readily detects SERBP1 on immunoblots (e.g. DOI 10.1093/nar/gkaa1189)<br>Anti-RPL5, Rb readily detects RPL5 on immunoblots (e.g. DOI 10.1158/0008-5472.CAN-18-2718)<br>Anti-RPS6, Rb readily detects RPS6 on immunoblots (e.g. DOI 10.1038/s41467-019-11227-4)<br>Anti-ATF-4, Rb readily detects ATF-4 on immunoblots (e.g. DOI 10.1038/s41467-019-08908-5)<br>Anti-GAPDH, Rb readily detects GAPDH on immunoblots of mouse heart, brain, and skin tissue per manufacturer<br>Anti-PELO, Mm readily binds PELO on immunoblots from mouse brain and thymus, and rat brain per manufacturer

Eukaryotic cell lines

Policy information about cell lines

Cell line source(s)	U2-OS cells (ATCC), F11 (Sigma)
Authentication	These cell lines were directly ordered from ATCC or Sigma, but not tested
Mycoplasma contamination	Cell lines were tested for Negative for Mycoplasma by PCR and found to be negative
Commonly misidentified lines<br>(See ICLAC register)	No commonly misidentified cell lines were used in this study

Animals and other organisms

Policy information about studies involving animals; ARRIVE guidelines recommended for reporting animal research

Laboratory animals	Mouse (Mus musculus), Swiss-Webster, male, 4-6 weeks old<br>Mouse, eEF2K KO, male, 4-6 weeks old
Wild animals	No wild animals were used in this study
Field-collected samples	No field-collected samples were used in this study
Ethics oversight	Institutional Animal Care and Use Committee of the University of Texas at Dallas protocol # 18-08

Note that full information on the approval of the study protocol must also be provided in the manuscript.
